# Editorial: Volume II: anti-inflammatory drug development focusing on immune mediated diseases

**DOI:** 10.3389/fphar.2026.1807303

**Published:** 2026-03-10

**Authors:** José Fernando Oliveira-Costa, Anubha Chaudhary, Suraj Singh Rawat, Amit Prasad

**Affiliations:** 1 Center for Infusions and Specialized Medicines of Bahia-CIMEB, Bahia State Health Department, Salvador, Brazil; 2 Immune Engineering Lab, School of Biosciences and Bioengineering, Indian Institute of Technology Mandi, Mandi, India; 3 Life Science Institute, University of Michigan, Ann Arbor, MI, United States

**Keywords:** anti-inflammatory drugs, drugs, immunology, inflammation, therapeutics

Dysregulated inflammation lies at the core of the pathophysiology of numerous immune-mediated diseases and represents a significant challenge for translational and clinical pharmacology. Despite advances in biologic therapies, small molecules, and targeted immune modulation strategies, gaps persist regarding sustained efficacy, long-term safety, and applicability in complex phenotypes. In this context, the second edition of this Research Topic expands upon the themes of the first volume (Oliveira-Costa and Prasad, 2024), bringing together contributions that reflect the therapeutic diversity and conceptual evolution of the field.

Pharmaceutical care is organized into three components: the Basic Component, targeting prevalent primary care conditions such as hypertension and diabetes (Ruas et al., 2025; Vieira, 2010); the Strategic Component, aimed at diseases of public health relevance, including tuberculosis, HIV, leprosy, and endemic diseases (Okamoto et al., 2022; Santana et al., 2021; Vieira, 2010); and the Specialized Component of Pharmaceutical Care (CEAF), responsible for high-cost, complex medications frequently used in chronic, rare, and autoimmune diseases, with access regulated by national protocols and tripartite funding (Ruas et al., 2025; Sopelsa et al., 2017; Vieira, 2010; Gottgtroy et al., 2025).

This edition aimed to bring together articles that discuss factors that could improve drug strategies for the management and treatment of immune-mediated diseases. The contributions presented here range from preclinical mechanistic studies to clinical trials and systematic reviews, highlighting novel molecular targets, emerging signaling pathways, and innovative therapeutic strategies.

An investigation into osteoarthritis caused by monosodium iodoacetate highlights the potential of Acetyl-11-keto-beta-boswellic acid (AKBA) as a treatment. Through the regulation of the HMGB1/TLR4/NF-κB and Nrf2/HO-1 pathways, AKBA illustrates how natural products can both reduce oxidative stress and inhibit inflammation. This study reinforces the potential of natural compounds as modulators of chronic inflammatory and oxidative stress processes, contributing to drug development with improved safety profiles (Abo-Zalam et al.).

The translational potential of anti-inflammatory strategies is also evident in clinical studies. In a randomized trial, Huang et al. evaluated the administration of flurbiprofen axetil before surgery significantly improved inflammatory regulation and pain management while also improving sleep quality, which is vital, yet often overlooked, recovery parameter. These findings confirm that preemptive NSAID therapy has physiological benefits and emphasize that administration timing is as critical as medication or drug selection. Consequently, integrating flurbiprofen axetil into preoperative multimodal protocols offers a practical and effective strategy to optimize patient outcomes following laparoscopic gynecological procedures.

In the context of refractory immune-mediated diseases, Kang et al. presented a systematic review and meta-analysis on the use Tocilizumab as an effective therapy for refractory noninfectious uveitis (NIU), associated with systematic disorders. The drug significantly improved visual acuity, reduced inflammation, and resolved macular edema, all while reducing the need for corticosteroids with minimal safety risks. The evidence suggests that IL-6 receptor inhibition is a robust alternative for patients who do not respond to standard immunosuppressants or TNF-α blockers, nevertheless, large-scale randomized trials are still required to standardize therapy procedures.

However, the role of IL-6 blockade extends beyond autoimmune disorders, it is increasingly relevant in metabolic-inflammatory context. Amer et al., demonstrated that blockade of IL-6 receptor can restore the activation of tissue-resident NK cells in the pancreas and ameliorate injury in an experimental mice model of metabolic dysfunction-associated steatohepatitis (MASH), highlighting the interconnection between systemic inflammation, tissue immunity, and metabolic diseases. Other contributions deepen the understanding of novel immunological targets. Matsushima et al., identified a compound, KIRA6 with anti-allergic activity, which effectively supresses the activation of human and rodent mast cells and basophils in both *in vitro* and *in vivo*. Utilizing knockout cell lines and kinase assays, the study demonstrates that inhibitory effect of KIRA6 occurs independently of IRE-1α. Instead, KIRA6 appears to target the Lyn/Syk dependent pathway, which is a critical driver of the allergic response, offering a promising perspective for the treatment of inflammatory allergic diseases. Complementarily, Sailer et al. explored the S1PR4 receptor-dependent effects of Etrasimod on human myeloid cell activation, contributing to the understanding of sphingosine-1-phosphate receptor roles in innate immune regulation. Etrasimod likely acts as functional antagonist/superagonist of the S1PR4 receptor, highlighting the crucial role of S1PR4 signalling in pro-inflammatory immune response, particularly in the context of inflammatory bowel diseases (IBDs).

Furthermore, cGAS- STING pathway has emerged as a critical regulator of sensing cytoplasmic DNA, eliciting immunological responses, and is linked to malignancies and multiple diseases. In a recent review, Luo et al., highlights the role of cGAS-STING pathway in inflammatory skin diseases, discussing therapeutic opportunities and challenges associated with modulating this cytosolic DNA-sensing pathway. This pathway facilitates intricate crosstalk between fibroblasts, keratinocytes, and various immune cells, ultimately triggering the chronic inflammation characteristics of STING-associated vasculopathy with onset in infancy (SAVI), Systemic lupus erythematosus (SLE), psoriasis and systemic sclerosis.

Ameer et al., provide a comprehensive review of dupilumab use in asthma, integrating mechanistic data, clinical outcomes, and cost-effectiveness considerations, reflecting the growing maturity of targeted biologic therapies. Dupilumab effectively disrupts the T2 inflammation by targeting IL-4 and IL-13 signaling, marking a significant advancement in precision medicine. The clinical and real-world outcomes support its superior efficacy in lowering asthma exacerbations, dependence on corticosteroids, and improving lung functions. It’s widespread effectiveness in treating atopic dermatitis and chronic rhinosinusitis highlights its systemic utility and favorable safety profile, which extends beyond respiratory treatment. Ultimately, dupilumab’s capacity to offer targeted, mechanism-based relief signifies a change toward individualized therapy that enhances long-term quality of life for patients with refractory inflammatory illnesses, even while pharmacoeconomic concerns still exist.

You et al., discuss the development of promising therapies for Enterovirus 71 (EV71) infection, highlighting how disease pathogenesis involves not only viral replication but also exacerbated inflammatory responses and cytokine storms. Despite geographic constraints for EV71 vaccines, a varied array of therapeutic strategies is emerging to target both the virus lifecycle and its lethal immunopathology. Current clinical strategies utilize immunoglobulin (IVIG) to neutralize the virus and Milrinone to regulate the cytokine storm, while emerging research highlights CCL3-mediated neutrophil recruitment as a potential target for mitigating brain damage. Experimental therapies include capsid function inhibitors such as pleconaril and the nucleoside analogue ribavirin, as well as neuroprotective medicines like minocycline, which traverses the blood-brain barrier to reduce inflammation. Moreover, highly specific antimicrobial peptides and bioactive natural compounds—such as magnolol, which suppresses ferroptosis, and etoposide, which obstructs the 2A protease—exhibit significant antiviral effects both *in vitro* and *in vivo*. Despite these advances, the lack of FDA-approved antiviral medicines for enteroviruses highlights the urgent necessity for high-throughput screening assays to convert these natural and synthetic candidates into standardized therapeutic therapies.

**FIGURE 1:**
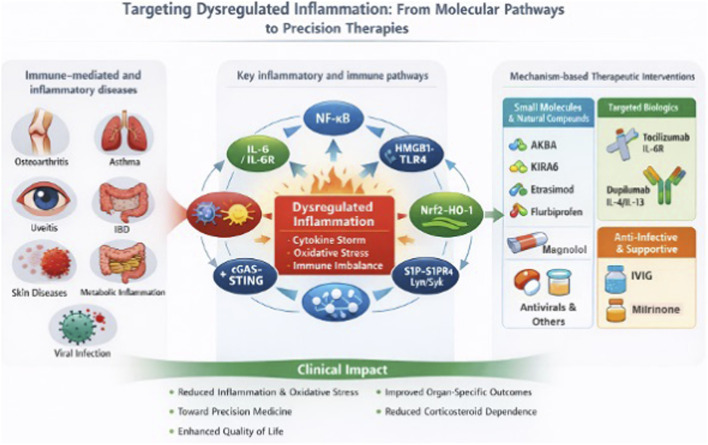
Commonly affected human organs by inflammatory disorders and their molecualr pathways and some of the suggested therapeutics.

The reviews included in this volume reinforce emerging trends in anti-inflammatory drug development which is summarised in Figure 1. Together, the articles in Volume II illustrate the dynamic nature of research in anti-inflammatory drug development, emphasizing the transition from broadly immunosuppressive approaches to more selective, mechanism-based strategies with higher potential for efficacy and safety. Similar to the first volume, this Research Topic underscores the importance of integrating basic research, translational pharmacology, and clinical evidence in addressing immune-mediated diseases, with the expectation that it will stimulate new investigations, interdisciplinary collaborations, and therapeutic advances that impact clinical management and patient quality of life.
